# Pre-operative anxiolysis in children through a combined pharmacological therapy with hydroxyzine and a non-pharmacological distraction technique with a clown (SONRISA): study protocol for randomised double-blind clinical trial

**DOI:** 10.1186/s13063-019-3906-2

**Published:** 2020-01-02

**Authors:** Esther Aleo Luján, Amanda Lopez-Picado, Ana Rivas, Belén Joyanes Abancens, Marina Laura Rodríguez Rojo, Patricia Fernández García, Carmen Soto Beauregard, Jaime Rodríguez Alarcón, Carlos González Perrino, Borja San Pedro de Urquiza, Eva Arias, Diamelis Rodriguez, Carmen Esteban Polonio, Maria José Torrejón

**Affiliations:** 1 Unidad de Cuidados Intensivos Pediátricos y Unidad de Recuperación Postanestésica, Servicio de Pediatría, Instituto del Niño y del Adolescente, Hospital Clínico San Carlos, Instituto de Investigación Sanitaria del Hospital Clínico San Carlos (IdISSC), C/ Profesor Martin Lagos s/n, 28040 Madrid, Spain; 2 Departamento de Pediatría, Facultad de Medicina, Universidad Complutense de Madrid, Madrid, Spain; 3 Unidad de Investigación Clinica y Ensayos Clínicos, Hospital Clínico San Carlos, Instituto de Investigación Sanitaria del Hospital Clínico San Carlos (IdISSC), Madrid, Spain; 4 Departamento de Enfermería, Facultad Enfermería, Fisioterapia y Podología, Universidad Complutense de Madrid, Madrid, Spain; 5 Servicio de Cirugía Pediátrica, Instituto del Niño y del Adolescente, Hospital Clínico San Carlos, Instituto de Investigación Sanitaria del Hospital Clínico San Carlos (IdISSC), Madrid, Spain; 6 Departamento de Pediatría, Facultad de Medicina Universidad Francisco de Vitoria, Madrid, Spain; 7 Servicio de Anestesiología y Reanimación, Hospital Clínico San Carlos, Instituto de Investigación Sanitaria del Hospital Clínico San Carlos (IdISSC), Madrid, Spain; 8 Supervisora de Enfermería del Servicio de Pediatría, Instituto del Niño y del Adolescente, Hospital Clínico San Carlos, Instituto de Investigación Sanitaria del Hospital Clínico San Carlos (IdISSC), Madrid, Spain; 9 Servicio de Análisis Clínicos, Hospital Clínico San Carlos, Instituto de Investigación Sanitaria del Hospital Clínico San Carlos (IdISSC), Madrid, Spain

**Keywords:** Anxiety, Surgery, Clown, Hydroxyzine, Randomised clinical trial

## Abstract

**Background:**

Surgery can generate significant stress and anxiety in up to 70% of the paediatric population. There are several pharmacological and non-pharmacological strategies to reduce pre-operative anxiety in children, however, they have several side effects and the available information about them is contradictory. The role of clowns and hydroxyzine in the management of anxiety is controversial, with some studies supporting and others contraindicating both strategies.

**Methods:**

We propose a randomised double-blind, controlled clinical trial that will evaluate the effectiveness of both interventions (hydroxyzine and clowns), alone or in combination, to reduce pre-operative anxiety (using the modified Yale scale of preoperative anxiety) in children aged 2–16 years undergoing outpatient surgery (*n* = 188).

Subjects will be randomised into two groups – (1) standard procedure (parental accompaniment) combined with placebo or (2) standard procedure combined with preoperative hydroxyzine. After randomisation, they will be divided by chance into two further groups, depending on the presence of clowns on the patient’s surgery day. Control of pre-operative anxiety will be determined in the four groups by a modified Yale scale of preoperative anxiety and cortisol levels. Compliance of children during induction of anaesthesia, time until anaesthesia recovery, presence of postoperative delirium and use of analgesia until discharge will be also assessed. For additional information, the children, parents and healthcare professionals involved in the study will complete a satisfaction survey.

**Conclusions:**

This study aims to gather evidence on which of these four therapeutic options achieves the highest reduction of pre-operative anxiety with the best safety profile to allow paediatricians and anaesthesiologists to use the most effective and safe option for their patients.

**Trial registration:**

ClinicalTrials.gov identifier: NCT03324828. Registered 21 September 2017.

## Background

Surgery can generate significant stress and anxiety in up to 70% of the paediatric population [1]. The need to have specific programmes to reduce the anxiety of children is of special interest if we consider the adverse effects of surgery associated with high preoperative anxiety (POA) [2]. High levels of POA are responsible for increased surgical morbidity [3], postoperative analgesia needs [4], and increased number of days of hospitalisation and rate of complications [1]. The management of POA in paediatric patients is a field under constant review, with the studies published to date having differing, controversial and non-conclusive results [5, 6].

**Fig. 1:**
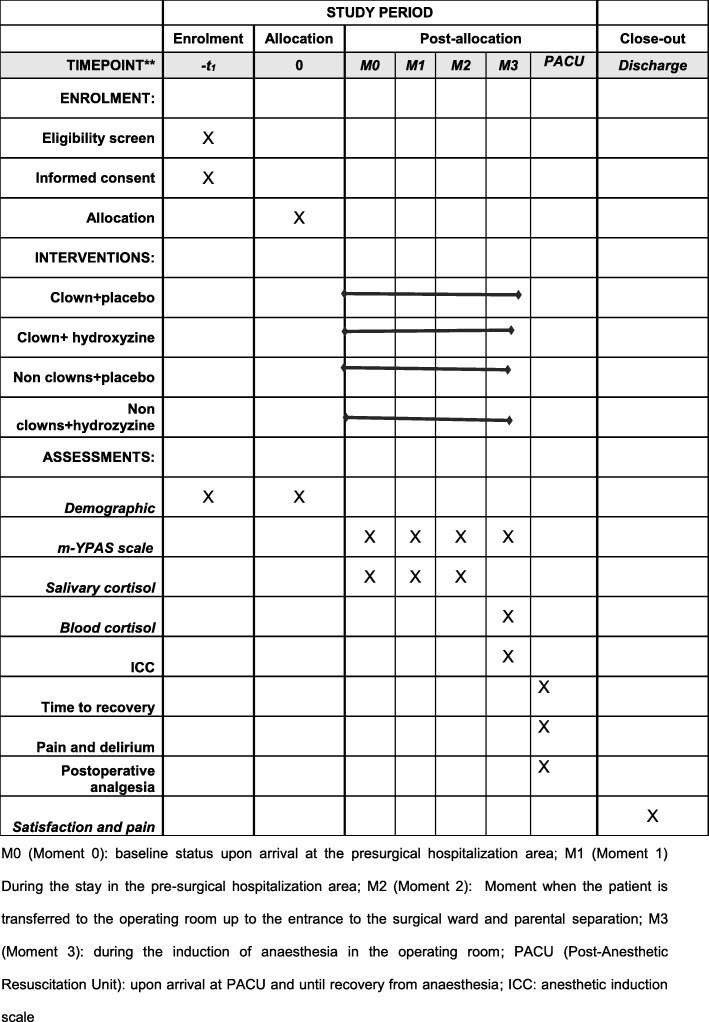
Example template of recommended content for the schedule of enrolment, interventions and assessments. M0 (Moment 0): baseline status upon arrival at the presurgical hospitalisation area; M1 (Moment 1): stay in the pre-surgical hospitalisation area; M2 (Moment 2): moment when the patient is transferred to the operating room up to the entrance to the surgical ward and parental separation; M3 (Moment 3): time during the induction of anaesthesia in the operating room; PACU (Post-Anaesthetic Resuscitation Unit): upon arrival at PACU and until recovery from anaesthesia. *ICC* Induction Compliance Checklist; *m-YPAS* modified Yale Preoperative Anxiety Scale

In order to reduce POA, strategic programmes that try to minimise the emotional impact have been designed. Some of these strategies are parental accompaniment during induction of anaesthesia [5, 6], sedative premedication [7] and distraction techniques [8, 9], including the presence of clowns [10] or music therapy [11].

Pharmacological studies performed with preoperative anxiolytic medication assess off-label drugs (such as clonidine) or drugs that require close monitoring and control measures (as midazolam) due to associated serious adverse events like delirium and respiratory depression [12, 13]. Hydroxyzine is an antihistamine with sedative properties approved for anxiolytic use both in Europe and the USA. Despite its widespread use in clinical practice, only few studies have assessed hydroxyzine’s effectiveness, most of which have been in the context of minor odontology interventions [14–16]. Furthermore, there are no clinical trials about its use for the management of POA in major outpatient paediatric surgery.

Studies related to non-pharmacological techniques for POA management have been published [5, 6, 8–11, 17] but results related to effectiveness of accompaniment and distraction are not confluents either [9, 17].

Humour and laughter have characteristics that could help reduce pain and stress but the information available is controversial; they seem to reduce anxiety in the area of hospitalisation prior to the operation room, but it has not yet been possible to demonstrate their benefit as anxiolytic therapy within the surgical area [18]. In a study by Vagnoli et al., 40 children aged between 5 and 12 years undergoing paediatric ambulatory surgery were randomised either to distraction and accompaniment by clowns and parents (experimental group) or to parental accompaniment only (control group). The experimental group showed significantly less anxiety during the induction of anaesthesia than the control group. According to a healthcare professional survey, professionals consider that clowns are beneficial for the children, however, most of the professionals believe that the presence of clowns adversely affects their work and would rather not to continue with this strategy [19]. In a subsequent study, Vagnoli et al. concluded that the combination of clowns and parental accompaniment during the preoperative stage achieved a higher reduction of POA than either parental accompaniment only or oral premedication with midazolam [20].

To summarise, the role of clowns and hydroxyzine in the management of anxiety remains unknown, with studies both positive for or against it. The present study aims to address this controversy.

The main objective of this clinical trial is to show whether the combination of hydroxyzine and distracting techniques with clowns have an additive effect for the control of POA. This objective will be evaluated by a specific validated scale (modified Yale Preoperative Anxiety Scale; m-YPAS) and determination of cortisol levels. In addition, the satisfaction of patients, parents and healthcare professionals with the strategy will be evaluated by a satisfaction survey.

## Methods/design

The study was approved by the appropriate Institutional Review Board and written informed consent was obtained from all subjects, legal surrogates, parents or legal guardians for minor subjects.

The trial was registered prior to patient enrolment at ClinicalTrials.gov identifier: NCT03324828 (Principal investigator: Esther Aleo Luján, Date of registration: October 30, 2017).

### Trial design

This is a unicentric, randomised, controlled clinical trial with parallel groups double blinded for pharmacological intervention. The CONSORT guidelines for randomised controlled trials will be used. This protocol also adheres to the SPIRIT guidelines. The study schedule of enrolment, interventions and assessments are included in Fig. 1.

### Population and setting

The study will be carried out in the Hospital Clínico San Carlos (HCSC), Madrid, Spain. Patients who will undergo paediatric ambulatory surgery will be included.

### Inclusion criteria

The inclusion criteria will be the following:

1. Children aged between 2 and 16 years old.
2. American Society of Anesthesiologists physical status classification grades I and II.
3. Informed consent (IC) signed by parents or legal guardians of the minors.
4. Specific informed consent for children aged between 12 and 16 years old.
5. No confirmed allergies to antihistamines.

### Exclusion criteria

The exclusion criteria will be the following:

1. Patients who had undergone previous surgery at age 2 years or older; when children undergo operations at an age younger than 2 years old, they do not remember the surgical experience and therefore the variables analysed are not influenced by this previous experience.
2. Patients with hypersensitivity to the active substance, to any of the excipients, to cetirizine, to other derivatives of piperazine, to aminophylline or to ethyleneamine.
3. Patients with porphyria.
4. Patients with diagnosed a prolongation of the QT interval.
5. Patients with risk factors for QT interval prolongation, including pre-existing cardiovascular disease, electrolyte balance disturbances (hypokalaemia, hypomagnesaemia), family history of sudden cardiac death, significant bradycardia and concomitant use of drugs with the potential to produce prolongation of the QT interval and/or induce Torsade de Pointes.

### Randomisation

Following signature of the IC by the legal guardians of the minors and/or the child’s agreement (if applicable), randomisation to treatment with/without hydroxyzine will be performed. Randomisation will occur in a 1:1 ratio in blocks of 8. The non-blinded nurse will randomise subjects with electronic case record form (REDCap). The sequence will be blinded to all team members.

Accompaniment by clowns (Dr. Sonrisas from Fundacion Theodora) will depend on the clown presence/availability on the day of the intervention. An alphabetical code will be assigned to patients assigned to clown accompaniment (A) or not (B).

### Intervention

The study participants will be allocated to one of these strategies:

- Group 1: Standard management consisting of parental accompaniment during the preoperative period, post-anaesthesia recovery area and up to hospital discharge.
- Group 2: Standard management combined with distraction and accompaniment by Dr. Sonrisas during the preoperative period, post-anaesthesia recovery area and up to hospital discharge. The clown will be present with the children during the post-anaesthesia period and up to discharge.
- Group 3: Standard management combined with pharmacological intervention (oral hydroxyzine 2 mg/kg masked with 5 ml of juice, administered at least 30 min prior to surgery).
- Group 4: Standard management combined with accompaniment and distraction by Dr. Sonrisas and hydroxyzine (as described in the previous groups).

All the patients will be filmed in order to later evaluate the patient’s state of anxiety by the m-YPAS scale. Cortisol levels in saliva (using Salivette®) and blood will be also analysed for all subjects.

### Blinding

In order to hide clown accompaniment to the evaluator of the m-YPAS, their appearance on the screen will be avoided and the film will be silenced. Subjects in whose film Dr. Sonrisas appears will be withdrawn from the study. The evaluation of all films will be done by the principal investigator.

Regarding hydroxyzine, the non-blinded nurse will administer 5 ml of juice to all subjects, mixed or not with hydroxyzine, depending on the treatment arm assigned.

### Treatment guideline

On the day of surgery, once the IC and, if applicable, child agreements have been signed and the selection criteria have been confirmed, the non-blinded nurse will randomise the subjects. Juice, with or without hydroxyzine, will be administered to subjects at least 30 min prior to the subject being transferred to the surgical ward.

POA evaluation will be performed at the following time-points:

- Time-point 0 (M0): Considered the baseline status because it is the moment when the patient arrives to the pre-surgical hospitalisation area prior to being in contact with any method to reduce POA. At this time-point, the investigators will record a film in order to evaluate the patient’s baseline state of anxiety by the m-YPAS.
- Time-point 1 (M1): During the stay in the pre-surgical hospitalisation area, at least 30 min after receiving the assigned strategy. At this moment, investigators will record a film to later evaluate the subject’s preoperative state of anxiety by the m-YPAS. The first Salivette® will be provided to the patient for the collection and determination of salivary cortisol. Salivette® will be collected before leaving the pre-surgical hospitalisation area.
- Time-point 2 (M2): Moment when the patient is transferred to the operating room up to the entrance to the surgical ward and parental separation. The investigators will record a film during the transfer to the operating room and up to the entrance to the surgical block to later evaluate a subject’s preoperative anxiety by the m-YPAS scale. At this time-point, the second Salivette® will be collected when the patient is placed in the operating room bed.
- Time-point 3 (M3): During the induction of anaesthesia in the operating room: The investigators will record a film during the induction of anaesthesia to later evaluate subject’s preoperative anxiety by the m-YPAS scale. At this time-point, the anaesthesiologist will complete the Induction Compliance Checklist and a blood sample (5 ml) will be obtained for cortisol analysis.
- Stay in Post-Anaesthetic Care Unit (PACU): Upon arrival at PACU and until recovery from anaesthesia, the variables about recovery from anaesthesia, pain, delirium, vital signs and postoperative analgesia will be collected.
- Stay in General Ward: at the time of hospital discharge, parents/legal guardians will complete satisfaction survey as will children aged 5 years or older. Healthcare professionals will complete this survey at the end of the study.

Subjects will exit the study at hospital discharge.

### Variables

#### Demographics

Age, sex, underlying pathology and the type of surgery will be collected.

#### Outcome measures

- POA assessment: All subjects included in the study will be filmed at four moments to later evaluate their POA by the m-YPAS scale.
- Saliva and blood cortisol determination: Patients’ saliva will be collected by Salivette® device at time-points M1 and M2. Likewise, a 5-ml blood sample will be obtained at time-point M3 to determine blood cortisol levels after the induction of anaesthesia.
- Induction Compliance Checklist: It will be performed by the anaesthesiologists in the operating room.
- Assessment of anaesthetic recovery with or without presence of delirium using PAEDS (PediatricAnesthesia Emergence Delirium Scale) [21] and assessment of post-anaesthetic recovery in PACU using Aldrete [22].
- Vital signs on arrival at PACU and every 30 min until the transfer to general ward: heart rate and respiratory rate.
- Pain Scales: Multidimensional pain scale for children under 3 years old, face pain scale for children aged 4–7 years and Visual analog scale for children older than 7 years old.
- Need of postoperative analgesia.
- Time to hospital discharge after leaving the operating room, in minutes.
- Evaluation on perceived healthcare quality: to fulfil this objective, a self-administered satisfaction survey will be completed by patients and parents/legal guardians after anaesthesia recovery and once hospital discharge is indicated. Healthcare professionals that usually attend children submitted to Major Outpatient Paediatric Surgery and that are involved in the study will complete the survey when the study is finished. The survey used is elaborated following the general criteria established by the Madrid Health Service for the elaboration of citizen satisfaction surveys [23]. Paediatric patients under 5 years old or older patients and parents/legal guardians with cognitive impairment or difficulty in understanding the language, will be excluded from this evaluation.

### Statistical analysis

#### Sample size

Anxiety in children will be evaluated through comparing m-YPAS score between the moment of induction of anaesthesia and the presurgical moment prior to the entrance to the operating room. The sample size calculated will allow detection of a difference of means of 14.3 points at m-YPAS between the group with parental accompaniment and hydroxyzine (mean: 18; standard deviation (SD): 13.5 points) compared to the group with parental accompaniment only (mean: 32.3; SD: 24.2 points) [4, 24]. With a sample size of 47 subjects in each group, we would have a power of 90%, with a level of significance of 0.025, to detect these differences. The final sample size would be 188 subjects (47 in each group).

#### Data analysis

The analysis will be performed by protocol and intention to treat. The qualitative variables will be presented with their frequency distribution. The quantitative variables will be summarised with their mean and SD. The quantitative variables that show an asymmetric distribution will be summarised with the median and interquartile range.

Comparison of the baseline characteristics of the four study groups will be performed. The association between qualitative variables will be evaluated by Pearson’s χ^2^ test or by Fisher’s exact test. For quantitative variables, means will be compared with Student’s *t* test for independent groups or with nonparametric test for the quantitative variables if they do not fit a normal distribution.

For the main outcome (POA) and secondary quantitative variables evaluated, a repeated measures analysis of the variance (ANOVA) will be carried out. The Bonferroni correction method will be used for multiple comparisons.

A comparison of means in each time-point and of the interaction between the dependent variable of the analysis (test score) and the study group (inter-subject factor) will be assessed. If any baseline characteristics between groups present clinically relevant differences, the model will be adjusted by those variables. Qualitative variables will be compared between groups using Pearson’s χ^2^ test. The relative risk with 95% confidence intervals will be calculated. If any of the baseline characteristics between groups presents statistically significant and/or clinically relevant differences between the four study groups, odds ratio (ORs) will be estimated using a logistic regression model. For all tests, a significance level of 5% will be accepted. The analysis of the data will be done by the statistical package STATA 12.0.

#### Safety

Adverse events will be carefully monitored throughout the study. All adverse events will be collected in the case report form for each subject, regardless of the causal relationship with study treatment. The investigator will notify, within 24 h, all serious adverse events, serious adverse reactions, unexpected adverse reactions, and serious and unexpected serious adverse reactions to the sponsor. If any information is not available at the time of notification, it will be completed within 7 calendar days with a follow-up report. The sponsor will promptly notify any information that could modify the benefit/risk ratio of the investigational drug or that determines changes in its administration schedule or in the performance of the trial.

#### Ethical approvals

The study has been approved by the Clinical Research Ethics Committee of the participating centre’s Hospital. The confidentiality of subject data will be maintained at all times in accordance with current legislation. All subjects and/or parents/legal guardians are informed about the study and will be asked for acceptance to participate in it. This study will be carried out following international ethical recommendations for conducting human research and clinical trials contained in the latest revision of the Declaration of Helsinki as well as those established in the Good Clinical Practice Guidelines and current legislation. All subjects will be supervised by qualified medical personnel during their participation in the study.

## Discussion

POA is observed amongst children during their stay in the operative room with a prevalence of 83.3% [23]. Adverse psychological and behavioural changes (decrease in children’s self-esteem and emotional well-being, increased anxiety, sleep disturbances and social isolation) have been observed in a rate of 25% in children during hospital stay and even at 1-year post-discharge [6]. A recent systematic review also identified negative psychological sequelae in children [18].

The search for an ideal premedication drug to reduce POA in children is ongoing. The drugs used as anxiolytics are not exempt of side effects and the studies about this issue are limited to dental procedures [14–16]. One study [15] evaluated hydroxyzine at doses of 0.15 mg/kg, yet the dose proposed in our clinical trial is of 2 mg/kg.

The role of clowns and hydroxyzine in the management of anxiety is controversial as there are studies both for and against them [18–20]. We have not found any study that determines the additive effect on anxiolysis of distracting techniques with clowns and pharmacological treatment, as we propose in our case.

The proposed study has some limitations. The clowns are not always at the hospital; therefore, assignment to this intervention depends on whether they are present or not on the day of surgery. In order to reduce this bias, the healthcare professionals that plan the patient’s surgery will not know when the clowns come to the hospital.

The main objective of this study is the evaluation of POA. Studies that evaluate POA are difficult to perform and interpret, mostly because of the difficulty in assessing anxiety attributable to the surgical act. According to some studies [3, 25], the moment with the maximum rate of anxiety and fear associated with the entire surgical procedure is during anaesthetic induction. Therefore, evaluating the child’s anxiety during anaesthetic induction is very useful to determine whether the strategies used in the presurgical period have been effective in reducing anxiety [26]. That is the reason why in the present study, POA is evaluated in both moments. Most studies on this topic use a presurgical anxiety rating scale, the m-YPAS [23, 24, 26, 27].

Additionally, cortisol levels in blood and saliva correlate with stress. Free fraction levels of cortisol in saliva are a faithful reflection of its serum levels and it increase in the same way when the patient is exposed to stressful situations that activate the axis. Therefore, in this study, in order to establish the effect of the different strategies to reduce POA, two different outcomes will be used, one objective (cortisol levels) and one subjective (m-YPAS).

In the reviewed studies, satisfaction surveys about healthcare quality are almost systematically carried out by parents [5, 6]. In addition, in many of them, the perception of the healthcare professionals involved is also requested, observing a disparate impact of the different POA management strategies depending on the professional questioned [9]. The present study will consider not only parents and professional opinion, but also children’s perception of the healthcare quality of the presurgical interventions to reduce POA.

If the hypothesis of this study is confirmed, the combination of clowns and hydroxyzine may be considered the most effective and safety option for the treatment of POA in children.

## Trial status

Subject recruitment started in 12th January 2018 and is ongoing, recruitment will be completed in 30th December 2019. Protocol version: 7 (1st April 2019).

## Supplementary information

**Additional file 1.** SPIRIT 2013 Checklist: Recommended items to address in a clinical trial protocol and related documents.

## Data Availability

Regarding sharing materials and managing of intellectual property, data will be available on request to the authors.

## Abbreviations

IC: Informed consent
PACU: Post-Anaesthetic Care Unit
POA: Preoperative anxiety

## References

[1] Kain ZN, Mayes LC, O'Connor TZ, Cichetti DV (1996). Preoperative anxiety in children. Predictors and outcomes. Arch Pediatr Adolesc Med.

[2] Banchs RJ, Lerman J (2014). Preoperative anxiety management, emergence delirium, and postoperative behaviour. Anesthesiol Clin.

[3] Chorney JM, Kain ZN (2009). Behavioural analysis of children’s response to induction of anesthesia. Anesth Analg.

[4] Kain ZN, Mayes LC, Caldwell-Andrews AA, Karas DE, McClain BC (2006). Preoperative anxiety, postoperative pain, and behavioural recovery in young children undergoing surgery. Pediatrics.

[5] Kain ZN, Mayes LC, Wang SM, Caramico LA, Krivutza DM, Hofstadter MB (2000). Parental presence and a sedative premedicant for children undergoing surgery. Anesthesiology.

[6] Kain ZN, Caldwell-Andrews AA, Mayes LC (2007). Family-centered preparation for surgery improves perioperative outcomes in children (A randomized controlled trial). Anesthesiology.

[7] Cox RG, Nemish U, Ewen A, Crowe MJ (2006). Evidence-based clinical update: does premedication with oral midazolam lead to improved behavioural outcomes in children?. Can J Anaesth.

[8] Patel A, Schieble T, Davidson M (2006). Distraction with a hand-held video game reduces pediatric preoperative anxiety. Paediatr Anaesth.

[9] Kerimoglu B, Neuman A, Paul J, Stefanov DG, Twersky R (2013). Anesthesia induction using video glasses as a distraction tool for the management of preoperative anxiety in children. Anesth Analg.

[10] Golan G, Tighe P, Dobija N, Perel A, Keidan I (2009). Clowns for the prevention of preoperative anxiety in children: a randomized controlled trial. Pediatr Anesth.

[11] Wang SM, Kulkarni L, Dolev J, Kain ZN (2002). Music and preoperative anxiety: a randomized, controlled study. Anesth Analg.

[12] Cao J, Shi X, Miao X, Xu J (2009). Effects of premedication of midazolam or clonidine on perioperative anxiety and pain in children. BioScience Trends.

[13] Dahmani S, Brasher C, Stany I (2010). Premedication with clonidine is superior to benzodiazepines. A meta analysis of published studies. Acta Anesthesiol Scand.

[14] Köner O, Türe H, Mercan A, Menda F, Sözübir S (2011). Effects of hydroxyzine-midazolam premedication on sevoflurane-induced paediatric emergence agitation: a prospective randomised clinical trial. Eur J Anaesthesiol.

[15] Faytrouny M, Okte Z, Kucukyavuz Z (2007). Comparison of two different dosages of hydroxycine for sedation in the pediatric dental patient. Int J Paediatr Dent.

[16] Torres-Pérez J, Tapia-García I, Rosales-Berber MA, Hernández-Sierra JF, Pozos-Guillén AJ (2007). Comparison of three conscious sedation regimens for pediatric dental patients. J Clin Pediatr Dent.

[17] Cuzzocrea F, Gugliandolo MC, Larcan R, Romeo C, Turiaco N, Dominici T (2013). A psychological preoperative program: effects on anxiety and cooperative behaviors. Pediatr Anesth.

[18] Sridharan K, Sivaramakrishnan G (2016). Therapeutic clowns in pediatrics: a systematic review and meta-analysis of randomized controlled trials. Eur J Pediatr.

[19] Vagnoli L (2005). Clown doctors as a treatment for preoperative anxiety in children: a randomized, prospective study. Peadiatrics.

[20] Vagnoli L, Caprilli S, Messeri A (2010). Parental presence, clowns or sedative premedication to treat preoperative anxiety in children: what could be the most promising option?. Paediatr Anaesth.

[21] Sikich Nancy, Lerman Jerrold (2004). Development and Psychometric Evaluation of the Pediatric Anesthesia Emergence Delirium Scale. Anesthesiology.

[22] Aldrete JA. The postanesthetic recovery score revisited. J Clin Anesth 1995:7:89. 91. 10.1016/0952-8180(94)00001-k 7772368

[23] MacLaren JE, Thompson C, Weinberg M (2009). Prediction of preoperative anxiety in children: who is most accurate?. Anesth Analg.

[24] Sadhasivam S, Cohen LL, Szabova A (2009). Real-time assessment of perioperative behaviors and prediction of perioperative outcomes. Anesth Analg.

[25] Aguilar Cordero MJ, Sánchez López AM, Mur Villar M (2014). Cortisol salival como indicador de estrés fisiológico en niños y adultos: una revisión sistemática. Nutr Hosp.

[26] Sadhasivam S, Cohen LL, Hosu L, Gorman KL, Wang Y, Nick TG (2010). Real-time assessment of perioperative behaviours in children and parents: development and validation of the perioperative adult child behavioral interaction scale. Anesth Analg.

[27] Kain ZN, Mayes LC, Cicchetti DV, Bagnall AL, Finley JD, Hofstadter MB (1997). The Yale Preoperative Anxiety Scale: how does it compare with a gold standard?. Anesth Analg.

